# Perceptions of roles of community healthcare workers in early childhood in Limpopo, South Africa

**DOI:** 10.4102/phcfm.v16i1.4412

**Published:** 2024-10-18

**Authors:** Aneesa Moolla, Lezanie Coetzee, Constance Mongwenyana, Anne Robertson, Gert Marincowitz, Michele Zuckerman, Fink Günther, Davidson H. Hamer, Aisha Yousafzai, Peter C. Rockers, Denise Evans

**Affiliations:** 1 Health Economics and Epidemiology Research Office, Faculty of Health Sciences, University of the Witwatersrand, Johannesburg, South Africa; 2 Swiss Tropical and Public Health Institute, University of Basel, Basel, Switzerland; 3 Clinical Department of Family Medicine, University of Limpopo, Polokwane, South Africa; 4 Department of Family Medicine, Faculty of Health Sciences, University of the Witwatersrand, Johannesburg, South Africa; 5 Department of Global Health, School of Public Health, Boston University, Boston, United States; 6 Department of Global Health and Population, Harvard T.H. Chan School of Public Health, Harvard, United States

**Keywords:** community healthcare workers, qualitative, early childhood development, child nutrition, immunisations

## Abstract

**Background:**

As part of the Reengineering Primary Health Care initiative, the South African National Department of Health (NDoH) has committed to expanding access to home-based care provided by community health workers. The NDOH also prioritised Community Health Workers (CHWs) in their agenda to improve child development outcomes in South Africa. However, there is limited research on CHWs’ experiences and knowledge of early childhood development.

**Aim:**

To explore CHWs’ motivation for work, their background, training and scope of work around Early Child Development (ECD).

**Setting:**

The study was conducted in Mopani District, Limpopo province, South Africa, in 2017.

**Methods:**

Five focus group discussions (FGDs) were conducted with 41 CHWs participating within a large cluster-randomised study. Data were analysed thematically using an inductive approach.

**Results:**

Community health workers’ motivation to work was influenced by personal experiences, community needs and community service. In terms of knowledge, CHWs indicated that a nutritious diet with extended breastfeeding, immunisations and preschool education is imperative for a child to thrive. The Road to Health Booklet, weighing scales and the mid-upper arm circumference tape were used as screening tools for ECD. Community health workers perceived their knowledge around ECD to be insufficient.

**Conclusion:**

Community health workers play a crucial role in healthcare; therefore, capacity development on ECD and the provision of ECD screening tools to optimise their under-five child visits are necessary.

**Contribution:**

This study will potentially contribute to the improvement of the CHW programme in ensuring that children under 5 years of age are holistically cared for to ensure that they thrive.

## Introduction

Community health workers (CHWs) have been part of the global healthcare system for over a 100 years and were first introduced in South Africa in the 1930s.^1^ Owing to the increasing burden of human immunodeficiency virus and acquired immunodeficiency syndrome (HIV/AIDS) during the 1990s, a large cohort of CHWs were recruited and trained to help ease the load on healthcare service provision.^2,3^ Since then, CHWs are increasingly shown to have the potential to improve the health status of the population, in particular those who are more disadvantaged or those living in hard-to-reach or inaccessible areas. Their involvement in mother and child programmes, HIV/AIDS and Tuberculosis (TB) programmes as well as chronic diseases and palliative care has been documented across many countries.^4,5^ Many studies^6,7^ have documented impressive impacts of CHW work, which includes improvements in disease prevention through health education, to significantly higher case finding than in standard care, better support for treatment adherence and increased control or cure rate, to better support for palliative care allowing patients to remain with their families at the end of their lives.

In South Africa, the roles of CHWs have broadened over the last decade to include other pertinent issues including early childhood development (ECD). Early childhood development refers to the critical physical, socioemotional, cognitive and motor development that a child experiences between birth and the age of 8 years, which significantly influences brain development. According to the South African Early Childhood Review (SAECR),^8^ of the 3.1 million children in South Africa aged between 3 and 5 years, only 69% were attending ECD programmes. Early childhood development programmes are intended to promote early emotional, cognitive, sensory, spiritual, moral, physical, social and communication development and early learning.^9^

The previous issue of the SAECR 2017^10^ estimated the under-six population of South Africa as being 6.2 million. Since then, Stats SA^8^ revisited and revised its midyear population estimates and subsequently updated the model used to calculate the population weights for its surveys, because it had come to attention that these estimates had been based on assumptions that were incorrect. The reweighted data show that there are many more young children than was previously thought. The under-six population was close to 7 million children in 2017, nearly a million more than expected. This is not the result of a sudden increase in births, but a result of the revised population model. According to the International Monetary Fund (IMF),^11,12^ South Africa also suffers from one of the highest Gini indices which measures wealth distribution. At least 13% of South African children are reported to be living in extreme poverty.^11,12^ Using Stats SA’s lower-bound poverty line, 53% of children (11.1 million) were poor in 2022 (up from 44% in 2019), and 38% (7.9 million children) were below the food poverty line, meaning that they were not getting enough nutrition. The study province, Limpopo, is also one of the poorest regions in South Africa. Poverty is notably high in the rural areas although urban poverty is also significant.^13^

Similarly, research in other low- and middle-income countries shows that children under the age of 5 years are unable to meet their cognitive and physical development potential because of poverty, poor health, inadequate early stimulation and undernutrition.^14,15^ Based on this evidence, ECD has become a priority strategy within South Africa, particularly in light of ensuring equity and high quality of care for children aged 0–5 years.^16,17^ Early childhood home-visiting programmes conducted by CHWs have subsequently proliferated in an effort to promote healthy child development and prevent neglect within at-risk families.^18,19,20^ The purpose of these home-visiting programmes is to provide caregivers with health information, nutritional and psychosocial support; support ECD and promote linkages and referrals to support services.^21^

It is widely believed that a well-functioning CHW platform has the potential to increase the coverage of a selection of interventions by at least 10%.^21,22^ This would translate into 34 800 additional lives saved over 10 years. Improvements to feeding practices would have the biggest impact. Promotion of vaccines accounts for 11% of the lives saved and the promotion of quality antenatal care for an additional 9%.^22^ In South Africa, CHWs play an important role in vertically delivered ECD programmes (frequently referred to as stand-alone, categorical, programmes) with many health promotion strategies also being delivered by them in order to increase access to health services for the general public. In these vertical disease programmes, CHW-led interventions are provided through delivery systems that typically have varied financial operations within the wider health system. This in turn impacts CHWs negatively because of these operations being additional programmes that they need to navigate over and above their current workloads.^22^

Thus, because of the lack of proper implementation of interventions, as well as inadequate monitoring and evaluations, not all vulnerable children are being reached.^20,23^ Despite being frontline workers who act as primary contacts to communities and families,^21,24,25^ CHWs face critical challenges that include poor resources, conflicting roles, remuneration issues and inadequate training.^18,26^ Despite these challenges, CHWs remain highly motivated and see their work as having a substantial impact on the health of the communities they serve.^26^ This study aimed to explore the perceptions of the roles of CHWs regarding early childhood in Limpopo, South Africa. The study also aimed to identify any training gaps that could be recommended to policymakers as criteria for CHW training that needs to be revised into current guidelines and brought into effect.

## Methods

### Study design

Data for this study were collected as part of a cluster-randomised controlled trial that aimed to evaluate the impact of an innovative package of early childhood interventions delivered by CHWs during routine home-based maternal and child health visits. This trial was done to test if a comprehensive package of early childhood interventions can be effectively integrated into existing CHW protocols in South Africa and if the intervention package can measurably improve early childhood health and development. The goals of these FGDs specifically were to get a better understanding of CHW day-to-day activities and experiences in Limpopo, specifically with regard to child development in order to enhance the interventions being designed.

We conducted a qualitative study comprising five focus group discussions (FGDs) with 41 CHWs in Limpopo province, South Africa.

### Setting

The study was conducted in Greater Tzaneen and Greater Giyani subdistricts within the Mopani District during 2017. Within Greater Tzaneen, there are 282 CHWs operating in 38 ward-based outreach teams (WBOTs), and in Greater Giyani, there are 268 CHWs operating in 30 WBOTs. The aim of the WBOTs is to improve access to primary health care (PHC) services including health promotion and disease prevention in South Africa. A WBOT includes a nurse who is an outreach team leader (OTL) supervising 6–10 CHWs. The team is linked to a facility and work within the municipal ward and offers promotive and preventive services to people at the household level.

### Study population

In mid-2017, South Africa’s total population was estimated at 57.7 million people, of whom 18.6 million were children under 18 years^27,28^ and about 8 million who were under 6 years. At least 787 469 of this under-6 children population resides in Limpopo.^29,30^ Furthermore, 115 735 children under the age of 5 years are located primarily in Mopani District, one of the most poverty-stricken areas in South Africa.^31^ Thus, given the large number of children under the age of 5 years residing in this province as well as the high number of CHWs working in this area, we chose to conduct the research in this area.

### Participant selection

Recruitment took place between January and February 2017. For the FGDs, we aimed to balance the number of CHWs of different ages and those who have worked as CHWs for different time spans. Potential participants were recruited purposively by sending out invitations to WBOT leaders in the Greater Giyani and Greater Tzaneen subdistricts. Ward-based outreach team leaders shared the invitation for the FGD with potential participants through clinic staff within the subdistricts. The study team was also invited by the WBOT to approach potential participants at the PHC clinics where they went in on certain days in order to give them a brief introduction to the study and ask if they were interested in participating. Those who indicated that they wished to participate were then screened by the study staff to ensure that they met eligibility criteria, which denoted that CHWs older than 18 years at the time of recruitment and had worked as a CHW for more than 3 months were deemed eligible. If eligible, CHWs were invited to attend an FGD, which took place at the clinic where their respective WBOT met weekly.

### Data collection

We obtained written informed consent from all who agreed to participate, after which these participants attended the FGDs. The discussion was led by the interviewer and research facilitator (who also took down notes) using an FGD guide that was devised by the research team and that incorporated themes around the CHW backgrounds, the structure of a typical work week and knowledge and perception around child development with a focus on under-five visits (see Appendix 1). Focus group discussions were conducted in the local languages (Xitsonga and Sepedi) by interviewers and facilitators not affiliated with the clinic and audio-recorded with participants’ consent. Focus group discussions were all conducted in private venues in the Greater Tzaneen and Greater Giyani subdistricts in Mopani District in Limpopo province, South Africa. There were between six and eight participants in all FGDs, which took 60–90 min each to complete.

Participant confidentiality is essential to the protection of human subjects and obtaining accurate information for the study. However, confidentiality can never be guaranteed, and this was relayed to participants, but assurance was given to participants that all study participants were briefed in their information sheets about confidentiality and were encouraged not to share information about the interview or participants outside of the FGD as this was considered to be confidential information. For all data collection methods, the participants were assigned a study identification (ID) number. Because we did not collect any personal identifiers, there was minimal risk of loss of subject confidentiality. The study database containing the processed interview responses was organised by the assigned study ID number and did not contain identifying information. Audio-recordings were transcribed and translated by the research team. The study research team conducted quality assurance measures to ensure that the transcripts were complete and accurate. The recordings, transcripts and study database were stored on password-protected work computers. The computers containing the study database were kept in a locked office when not in use by the study team. All database files were password protected, and only study staff had access to the files. Hard copy documentation was stored in locked cabinets in the work offices. Upon completion of the final interviews, hard copies of documentation (interview guides, information sheets and consent forms) were scanned and saved on secure computer servers only accessible to the research team. The electronic copies of the originals will be kept for 7 years after study completion before destroying them or until the data are published. Audio files were destroyed within 1 month of completing the analysis of the interview data.

### Data analysis

Audio files were transcribed verbatim, translated into English, checked for accuracy and completeness by the interviewers and research facilitators within the study team and then entered into NVivo for thematic analysis (NVivo 11; QSR International Pty Ltd.). Transcripts were read, re-read and summarised by two researchers from the study team in order to identify the initial themes and develop a codebook. Four transcripts were completely coded by two researchers of the study team and compared for intercoder reliability. The researchers established a preliminary codebook framework a priori and then followed an inductive, grounded theory analysis process, allowing new codes to arise spontaneously. The codebook was updated iteratively in regular feedback sessions with the coding team. The interview transcripts were coded using the same ‘master’ codebook to allow for comparability across interview types. If changes were made to a version of the codebook, then all transcripts were recoded by the researchers in the study team in order to ensure consistency of data analysis.

### Ethical considerations

An application for full ethical approval was made to the Human Research Ethics Committee (Medical) and ethics consent was received on 22 June 2016. The ethics approval number is M160251.

## Results

Of the 41 CHWs who participated in the FGDs, most (76%) were between 31 and 50 years of age (*n* = 31) and grew up in the community where they are currently working (*n* = 33; 80.4%; Table 1). The highest education status reported by CHWs were Grade 11 (*n* = 13) and Grade 12 (*n* = 21), with a few CHWs reporting having higher levels of education, which included training in ECD and certificates and/or diplomas relevant to their field of work.

**TABLE 1:** Community health worker demographics.

Descriptive variable	CHWs (*n* = 41)		Greater Giyani (*n* = 17)		Greater Tzaneen (*n* = 24)	
	*n*	%	*n*	%	*n*	%
**Age (years)**						
18–25	1	2.4	1	5.8	0	0.0
26–30	7	17.0	4	23.5	3	12.5
31–40	12	29.2	1	5.8	11	45.8
41–50	19	46.3	11	64.7	8	33.3
51–60	2	4.8	0	0.0	2	4.8
**Years working as a CHW**						
1–5	23	56.1	5	29.4	18	75.0
6–10	7	17.0	4	23.5	3	12.5
11–15	9	21.9	7	41.1	2	8.3
16–20	2	4.8	1	5.8	1	4.1
**Grew up in the community where they are working**						
Yes	33	80.4	15	88.2	18	75.0
No	8	19.5	2	11.7	6	25.0
**Marital status**						
Single	23	56.10	4	23.5	19	79.1
Married	14	34.1	10	58.8	4	16.6
Divorced, widowed or separated	4	9.7	3	17.6	1	4.1
**Highest level of education**						
Grade 10	2	4.8	1	5.8	1	4.1
Grade 11	13	31.7	3	17.6	10	41.6
Grade 12	21	51.2	11	64.7	10	41.6
Level 4 (ECD training)	2	4.8	0	0.0	2	8.3
Certificate	1	2.4	1	5.8	0	0.0
Diploma	2	4.8	1	5.8	1	4.1

CHW, community health worker; ECD, early child development.

### Community health worker context

#### Factors that motivate community health workers to do their work

**FIGURE 1:**
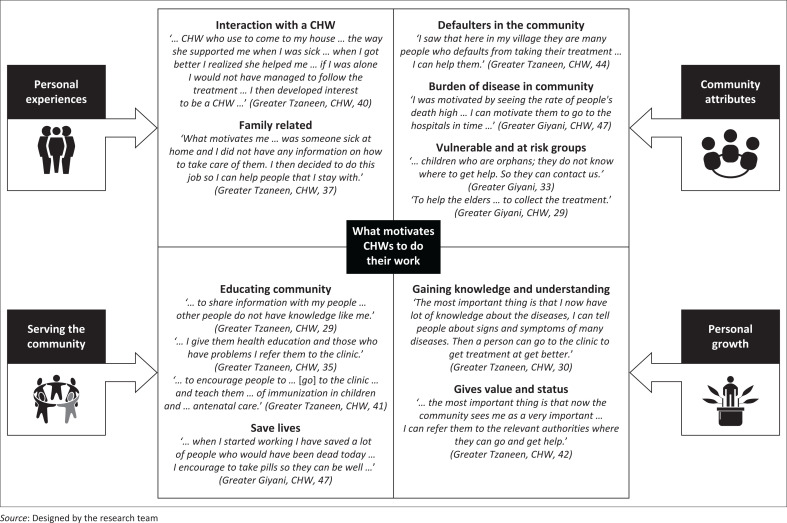
Main motivators for community health workers (CHWs), identified from five focus group discussions (FGDs) with CHWs, included experiences, community needs, personal growth and serving the community.

Most CHWs reported that their motivation to work (Figure 1) was influenced by multiple factors, which included: (1) personal experiences, for example, experiences with helpful CHWs during their youth, as well as being able to personally help sick family members; (2) recognising community needs, for example, high burden of disease, medication defaulters and vulnerable groups, as well as being able to contribute by reaching these populations in need; (3) personal growth and status, for example, knowledge acquisition and recognition within the community and lastly; (4) educating the community and referring individuals to the clinic if needed and ultimately being able to save lives through their work.

Community health workers strongly identified with the need for care and support within their communities and shared the hope that their work will achieve better health and a better quality of life for those they serve. Identifying with the need for care and support within their families and community, CHWs can be seen to take responsibility for their community’s health with the hope of seeing them prosper. Many participants indicated that they felt excitement at seeing the effects that their work had on the well-being of people they helped and who ended up displaying increased awareness of the health matters that they were being educated on. Participants also unanimously agreed that the community’s understanding of health messages stemming from the health talks that they as CHWs conduct motivates them personally to continue with their day-to-day work, which also includes making appropriate referrals to the clinic in order to reduce the burden of disease within their respective communities (Figure 1):

‘[*S*]o now it excites me because I see they are well and are living just like everyone …’ (Greater Giyani Limpopo, CHW, Female, 45 years old)

‘I’m also excited that people are now highly aware …’ (Greater Giyani Limpopo, CHW, Female, 47 years old)

### Community health worker knowledge and perceptions on factors impacting early child development

**FIGURE 2:**
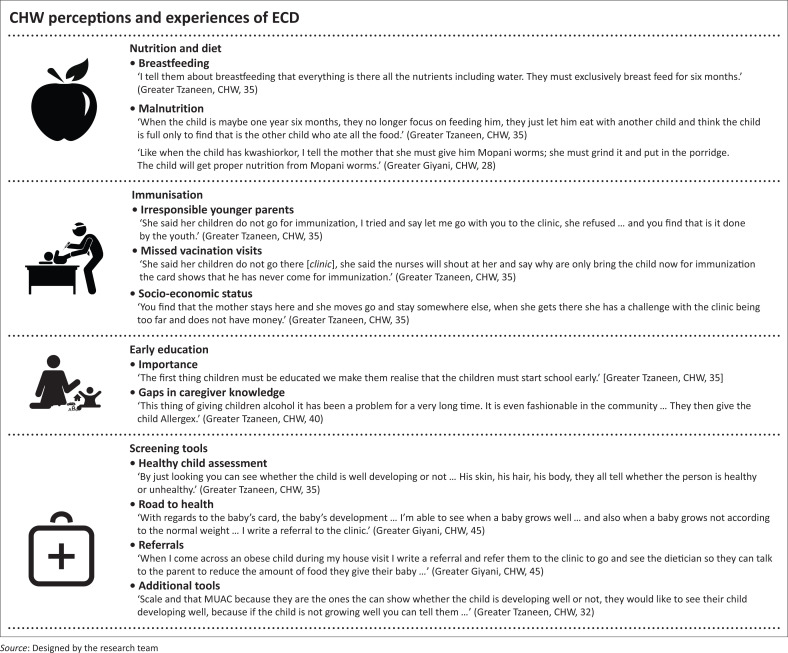
Community health worker perceptions and experiences of early child development.

Despite the limited training received, CHWs were knowledgeable in that a nutritious balanced diet with extended breastfeeding of more than a year, immunisations and early preschool education is imperative for a child to thrive (Figure 2).

#### Nutrition and diet for children under the age of 5 years

Most CHWs reported that they encourage mothers to breastfeed exclusively for 6 months and then for an extended period of up to 2 years because it is the most nutritious food for children under the age of 5 years and crucial for healthy physical development. Participants reported that they also advise mothers that solid food given too early could be harmful and potentially lead to poor health outcomes in children (Figure 2):

‘When you give him solid food his stomach will grow big; others can even die.’ (Greater Giyani Limpopo, CHW, Female, 43 years old)

‘They should breastfeed for at least two years.’ (Greater Tzaneen Limpopo, CHW, Female, 32 years old)

Some CHWs indicated that often young children (< 5 years) are left to feed themselves, and this is perceived to also lead to malnourishment in these young children as older children ate a greater portion of the food. Other participants also reported that they advise caregivers to feed children Mopani worms and porridge when they show symptoms of stunting or malnutrition and/or kwashiorkor because of the health benefits that these foods provide (Figure 2). Community health workers felt that they needed to have more information and training on how to deal with these situations.

#### Immunisation

As noted, a perception of a healthy child is one who has an up-to-date immunisation schedule. However, CHWs often find that they are unable to check on a child’s vaccination schedule status as a result of parents being unwilling to produce the Road to Health Booklet (RTHB) because of a variety of personal reasons, including disclosure of mother’s HIV status in this booklet. The RTHB is a health booklet, which the clinic provides for each child and contains a summary of the child’s health during the first 5 years of life.^24^ There also exists a perception among CHWs that younger parents can be irresponsible in terms of vaccinations and generally refuse to accept that vaccines offer disease protection, while older parents are more responsible and take their children to the clinic to be vaccinated. Many of the parents who claim to have lost the RTHB are also adamant that vaccines are not as important as society perceives them to be (Figure 2).

In contrast, there are parents who have missed vaccination visits and then feel afraid to return to clinics because of their experiences of nurses being verbally abusive if they missed clinic visits and then returned later (Figure 2). Importantly too is the socioeconomic interplay, which denotes that some mothers just do not have the financial resources (money for transport, loss of daily work income) needed to take their children to be immunised (Figure 2). An additional challenge noted by CHWs is that they are working with a mobile community consisting of people who move from one village to another, and this makes it difficult to trace parents if children have missed vaccinations. Thus, CHWs felt that they needed to have support, as well as more information and skills training on managing their relationships with caregivers and dealing with challenging communication situations.

#### Early childhood development screening methods and tools used by community health workers

Participants further explained that the RTHB served as the most important tool for them to monitor and assess the health of children under 5 years, which includes changes in height or weight that are used to validate the children’s physical assessments. Measurements in the RTHB are used to track height for age and weight for age over time, and if children do not meet recommended scores,^25^ then CHWs refer them to the nearest clinic for further testing. Furthermore, if children are weighed and shown to be overweight according to RTHB measurements, then they are referred to a dietitian based at the closest local clinic for assistance with weight management (Figure 2).

Although the RTHB serves as the most important tool that CHWs use during the under-five home visits, participants highlighted that there are other tools that they use when conducting home visits. This includes scales for weighing the children and tape to measure the mid-upper arm circumference (MUAC), both used to measure the physical growth of the child (Figure 2).

Participants reported that when caregivers feel that they are providing a nurturing environment for their children, then they are eager to have their children’s development monitored as they perceive these measurements to be reflective of their efforts. Similarly, caregivers whose babies do not look physically healthy are generally hesitant to have measurements (e.g. height, weight, MUAC) of their babies taken. Participants indicate that they start off by using their experience and discretion to assess the physical appearance of children, as a proxy of the child’s health. If a child appeared to be physically healthy with no signs of any diseases, then the child was deemed to be healthy (Figure 2).

Community health workers also use the RTHB to check various measurements or tools and/or assess whether children’s gross motor skills are developing adequately. The RTHB thus serves as a guidance tool and gives direction to both CHWs and parents on which areas to focus on with regard to healthy child development as well as what to do if a child is unwell and has symptoms that have serious impacts, such as vomiting:

‘Let’s say the child is vomiting; I check inside the card; it explains that I need to take the child to the clinic.’ (Greater Giyani Limpopo, CHW, Female, 24 years old)

Results show that the RTHB has the potential to be misused by caregivers, which CHWs indicate is happening more frequently at present. Community health workers denote that caregivers use the RTHB as a means to an end with regard to the attainment of the South African Social Security Agency (SASSA) child support grant for lower-income households to assist parents with the costs of their children’s basic needs. These parents often use the grant (ZAR 440 or $31 per month, per child) to feed the entire family, and it is within these families that CHWs are starting to notice malnourishment among children:

‘You find that the whole family depends on the social grants of one child.’ (Greater Tzaneen Limpopo, CHW, Female, 29 years old)

In summary, CHWs unanimously agreed that the most important aspect to focus on in the RTHB when doing under-five visits is to check whether the child is developing according to the development phases in the RTHB and is up-to-date with immunisations, vitamins and deworming. If the child is lacking in any of these aspects, CHWs refer the child to the clinic for the appropriate treatment that is lacking:

‘We have to check whether the child received all the vaccines, the vitamin, deworming, to see whether that road to health card is up to date.’ (Greater Tzaneen Limpopo, CHW, Female, 35 years old)

#### Gaps in caregiver knowledge and training

Community health workers showed concern about gaps in caregiver knowledge with regard to substances and medication being given to very young children. Community health workers described how sometimes caregivers give children alcohol and medication to make them sleep. This is frequently done with children who had a tendency to cry, so caregivers tend to adopt these dangerous strategies to calm frustrated children without understanding the risks that these substances pose to young children (Figure 2). Community health workers indicated a need for more skills and training to be able to help caregivers understand the detrimental effects of their actions. Community health workers explained that even though they have had basic training, it is crucial that they receive additional updated and more comprehensive training on child development in order for them to continue to effectively educate caregivers during under-five visits. Training requests for mother and child nutrition, ECD educational material, maternal health and antenatal care were also highlighted as specific training needs because these communities have an increased risk of morbidity and mortality:

‘Many people do not care about going for antenatal care and much death that we found is maternal death.’ (Greater Giyani Limpopo, CHW, Female, 32 years old)

Of these requests, child nutrition was of great concern as participants deemed it to be an area where they lacked in-depth knowledge with regard to dietary information for introducing solids for babies at 6 months:

‘There are those ones who give the baby breasts milk and mix with formula, they do not know, so we want to teach them….’ (Greater Tzaneen Limpopo, CHW, Female, 40 years old)

‘After that six months what type of food should the child eat….’ (Greater Tzaneen Limpopo, CHW, Female, 32 years old)

The majority of CHWs specified that they would like in-depth training on the RTHB around what it entailed:

‘This thing of RTHB we just come across it and got trained for 2 or 3 days but they did not train us well.’ (Greater Tzaneen Limpopo, CHW, Female, 41 years old)

## Discussion

Early childhood development in low- and middle-income countries and similarly in South Africa is noted to be a multidimensional public health issue^32^ influenced by a list of factors spanning from malnutrition to a lack of responsive caregiving. This study looked into the background of CHWs, what motivated them to become CHWs and what their scope of work around ECD entailed. The focus on children under the age of 5 years is a particular strength of this study as literature on this age group is scarce.^32^

There was a variation in the demographics of the CHWs between the two subdistricts, but there is no evidence to indicate that mainly older and more experienced CHWs also reside in one subdistrict only. We found that CHWs find fulfilment in serving their communities and contributing to lifting the burden of disease in these communities through education, supporting the vulnerable groups like teenage mothers and orphans and strengthening the health system through being the link between clinics and the community.

In terms of perceptions of the most important aspects of ECD, CHWs focussed mainly on food and indicated that a nutritious balanced diet with extended breastfeeding, followed by a completed list of immunisations and preschool education, is imperative for a child to thrive. Community health workers recommend extended breastfeeding because they feel that it is the most nutritious food for children under the age of 5 years and crucial for healthy physical development. This is partially supported by the WHO, which states that promoting breastfeeding at birth enhances the child’s cognitive and emotional development.^33^ Extended breastfeeding is supported by other studies that show it has long-term benefits.^34,35,36,37^ Mothers are also further encouraged to exclusively breastfeed children for 6 months as research^38,39^ indicates that, food given too early could pose health risks, some life threatening to the child. For children who were not developing well physically, CHWs encouraged the intake of Mopani worms, which is evidenced in other studies to be very nutritious.^39^ Mopani worm is a species of emperor moth, which is native to the warmer parts of Southern Africa. It is a large edible caterpillar that feeds primarily, but not exclusively, on Mopani tree leaves.^40^

On a limited note, CHWs noted that talking with children and caring for them were also important aspects of a child’s development. The focus on healthy physical development is also deemed to be one of the most crucial aspects of a child’s development by CHWs. The RTHB, weighing scales and the MUAC tape served as their most important screening tools. Ultimately though, CHWs perceived their own knowledge around ECD to be insufficient in terms of their in-depth knowledge about each of these important aspects of ECD. Thus, there were aspects not discussed in FGDs that related to other factors that could impact on ECD such as the mental health of the caregiver. There are also no clear guidelines as to what exactly CHWs need to cover in their under-five home visits, except for checking in on what the RTHB denotes as satisfactory ECD.

In terms of interactions with caregivers, CHWs indicated that they encourage caregivers to ensure that children receive all immunisations and refer children to the clinic immediately if there is evidence that the child’s vaccination schedule is incomplete. However, many CHWs note that they are unable to ascertain some children’s vaccination schedule status because of parents being unwilling to produce the RTHB because of the protection of their HIV status. There also exists a perception among CHWs that younger parents are reluctant to vaccinate their children, which is evidenced in literature.^41^ An incomplete vaccination schedule was also related to missed clinic visits and participants feeling afraid to return to the clinic because of being reprimanded by nurses. Similar to our findings, Oku et al. found that impolite behaviour of health workers towards teenage mothers was the reason for young mothers not bringing babies for vaccinations.^42^ Other reasons for missed vaccination visits include the high cost of transport, busy work schedules, lack of knowledge around vaccination schedule, inefficient communication between health personnel and caregivers, lack of trust in the public health system, geographic inaccessibility and preferences for traditional medicine.^42,43^

Caregivers are also encouraged to send their children early to crèche in order for the child to develop new skills and gain new knowledge, which aligns with findings that playgroups are a promising low-cost ECD intervention.^19^ In summary, nutrition, early childhood play stimulation and psychosocial activities have all been shown to have a direct impact on all aspects of the development of a child.^44^ This multifaceted notion needs to be addressed through interventions, as caregivers need to be: (1) offered nutritional advice that they can incorporate into the food regimen of their child, and (2) introduced to developmental activities that they themselves could perform with their children in cases where early education is not an option because of their socioeconomic status. This has been shown to be successful in other CHW-delivered parenting interventions as shown in a collation of the outcomes of CHW interventions by Viswanathan et al.^45^

In terms of tools, the RTHB serves as the main tool to assess physical development in children in terms of gross motor development milestones. However, there is a challenge to ascertain health-related information such as missed vaccination and clinic visits because some caregivers fail to produce the RTHB. Findings further show that CHWs do not always have essential tools like weighing scales or MUAC tape during their under-five visits. This shows that the CHWs need to be equipped with all necessary tools in order for them to be able to do their house visits efficiently. Caregivers also need to be encouraged to produce the RTHB so that CHWs could assist with advice and care specifically related to their child.

Despite the fact that the CHWs enjoy working with the community because they notice evidence of the impact of their knowledge and training on child rearing, they would like to offer more to the communities that they serve. It was clear that training across different WBOTs was not standardised or consistent. Training gaps were identified in: (1) mother and child nutrition, (2) ECD educational material, and (3) maternal health and antenatal care. Additionally, findings show that there is also a need for more training on how to assess the health of the children under 5 years.^21^

The breadth of public health issues addressed by the 2018 United Nations Sustainable Development Goals (SDGs)^33^ affects both maternal and child health in that the SDGs include specific ECD goals like improving access to ECD care and preprimary education (Target 4.2: By 2030, ensure that all girls and boys have access to quality ECD, care and preprimary education so that they are ready for primary education).^46^ Policy advances globally are indicated to be now putting ECD at the centre of efforts in order to address these critical factors.^47^ Addressing these issues is thus essential to align with SDGs. Therefore, it follows that it is crucial to learn the current understanding and perceptions of ECD among different healthcare workers in South Africa. Exploring the barriers and facilitators of the implementation of ECD practices within the community could inform the prioritisation of ECD within our own national health agenda and policies. A limitation of this study is the small sample size, but this study provides a valuable platform to showcase the work done by CHWs.

In many low-income countries, the CHW platform has been very patchy with inadequate training, inadequate support and supervision, uncertain funding and low morale among CHWs. This weak system then leads to underperformance and disappointing results.

## Conclusion

Community health workers play a substantial role in both the healthcare system and their communities, particularly with children under the age of 5 years. It is therefore crucial that CHWs be capacitated with comprehensive training on ECD and be equipped with appropriate tools to optimise their under-five visits within their communities. This study highlighted that communication with caregivers is challenging; thus skills training on management of relationships is critical to ensure that caregivers share the required information. Although this was intuitively expected, there were important findings relating to the work they do for ECD and maternal health in their communities.
